# Leveraging microphysiological systems to expedite understanding of host–parasite interactions

**DOI:** 10.1371/journal.ppat.1013088

**Published:** 2025-04-24

**Authors:** Maria Zorrinho-Almeida, Jorge de-Carvalho, Maria Bernabeu, Sara Silva Pereira

**Affiliations:** 1 Católica Biomedical Research Centre, Católica Medical School, Universidade Católica Portuguesa, Oeiras, Portugal; 2 Gulbenkian Institute for Molecular Medicine, Oeiras, Portugal; 3 EMBL Barcelona, Barcelona, Spain; Joan and Sanford I Weill Medical College of Cornell University, UNITED STATES OF AMERICA

## Abstract

Microphysiological systems (MPS) replicate the dynamic interactions between cells, tissues, and fluids. They have emerged as transformative tools for biology and have been increasingly applied to host–parasite interactions. Offering a better representation of cellular behavior compared with traditional *in vitro* models, MPS can facilitate the study of parasite tropism, immune evasion, and life cycle transitions across diverse parasitic diseases. Applications span multiple host tissues and pathogens, leveraging advanced bioengineering and microfabrication techniques to address long-standing knowledge gaps. Here, we review recent advances in MPS applied to parasitic diseases and identify persisting challenges and opportunities for investment. By refining these systems and integrating host multicellular models and parasites, MPS hold vast potential to revolutionize parasitology, enhancing our ability to combat parasitic diseases through deeper mechanistic understanding and targeted interventions.

## Microphysiological systems as a novel alternative to tackle old questions

A microenvironment is a local volume where different cell types interact with each other and with mechanical elements, establishing complex relationships that result in emerging behaviors and function [1]. Microphysiological systems (MPS) are multi-cellular *in vitro* systems where cells are exposed to a microenvironment that replicates specific characteristics of a tissue or organ. They allow assessment of cell physiology and multiscale cellular interactions and, ultimately, help to identify disease mechanisms and novel therapeutics. The use of MPS shows a strong potential for the study of host–parasite interactions, as these interactions are heavily influenced by the specific physiological conditions of the host tissues. Host–parasite relationships are dynamic, involving the interplay between the parasite’s survival mechanisms and the host’s immune response. For instance, several parasites display tropism to specific tissues, and the efficacy of both drug treatment and host responses can vary between them, creating parasite reservoirs [2]. Recreating specific microenvironments can expedite our understanding of how different tissues of the host are colonized, how immune responses are suppressed, and how parasites respond to various stimuli and environmental cues.

Progressive advances in bioengineering and microfabrication have made MPS a promising alternative to either bridge traditional *in vitro* systems with *in vivo* models or fill gaps from *in vivo* work [3]. Several microfabrication techniques are available to generate tailored MPS, with different levels of accessibility, scalability, dimensionality, and resolution (Box 1). Altogether, they are priming seminal discoveries in non-communicable diseases, such as reconstituting tumor pathophysiology with dynamic extracellular matrix stiffnesses, which determine metastatic behavior [4], or expediting tissue regeneration, which can offer alternatives to transplantation [5]. In parasitology, there has been considerable effort to adapt and apply these technological advances to tackle previously intractable questions. Here, we will review current uses of microenvironments for the study of host–parasite interactions and identify key knowledge gaps to which these tools could contribute to. We will focus on selected pathogenic endoparasites of human and animal relevance, including apicomplexans, kinetoplastids, and nematodes. Summarized life cycles of the parasites described in this review are provided in Box 2.

## Models of the blood-brain barrier

**Fig 1:**
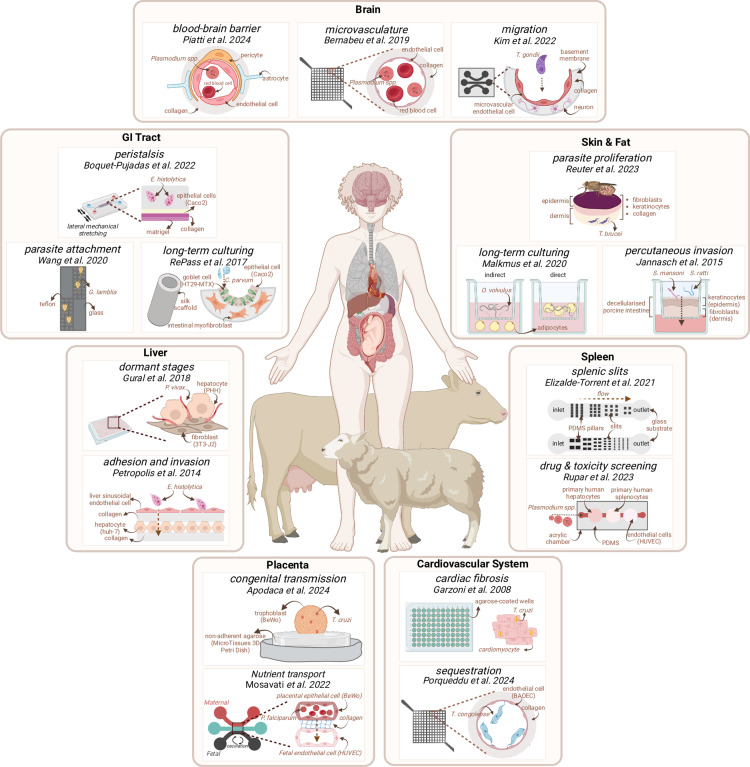
Body distribution of selected MPS used in the field of host-parasite interactions research. Brain: a blood-brain barrier (BBB) model using a collagen matrix, astrocytes, pericytes, and endothelial cells has been developed for the study of malaria-induced BBB breakdown [16]; a grid-like human brain microvasculature model was developed to study *Plasmodium*-infected red blood cell sequestration to the vascular endothelium [17]; a microfluidic model of toxoplasmosis-associated transendothelial migration with neuron co-culture [18]. GI Tract: a stretchable model was developed to study the effect of peristalsis on *Entamoeba histolytica* adhesion to the intestinal epithelium [19]; a long-term culture system was developed to support *Cryptosporidium parvum in vitro* growth [20]; and a multi-patterned model simulates the biomechanics of parasite attachment during giardiasis [21]. Skin & Fat: a compressed skin model was developed to study *Trypanosoma brucei* proliferation in the skin [22]; a decellularized porcine intestine model was developed to study *Schistosoma mansoni* and *Strongyloides ratti* percutaneous invasion [23] and an adipocyte model was created to support long-term culturing of *Onchocerca volvulus* [24]. Liver: a 3D liver platform to study *Plasmodium vivax* liver development, including the hypnozoite dormant state [25] and a sandwich-model to study the determinants of *E. histolytica* adhesion and invasion of the liver [26]. Spleen: an acellular model to study splenic pitting during *Plasmodium spp.* [27], and a multi-organ-on-a-chip model to study antimalarial efficacy [28]. Placenta: a spheroid placenta model allowed study of Chagas disease congenital transmission [29] and a microfluidic model allowed study of nutrient transport in pregnancy-associated malaria [30]. Cardiovascular System: a spheroid model of Chagas fibrosis [31] and 3D bovine microvessel model of the aorta to study *Trypanosoma congolense* sequestration [32] were developed. Created with Biorender (https://BioRender.com/e28s661).

The blood-brain barrier (BBB) serves as a shield to prevent harmful substances and pathogens from entering the brain, as well as to maintain homeostasis. This key function arises from the crosstalk between endothelial cells, pericytes, and astrocytes. BBB breakdown plays a crucial role in the development of several parasitic diseases, including cerebral malaria [6], sleeping sickness [7], animal African trypanosomiasis [8], cerebral theileriosis [9,10], babesiosis [11], *Acanthamoeba* granulomatous encephalitis [12,13], and *Balamuthia* amebic encephalitis [14]. The mechanisms by which some parasites can cross the BBB into the Central Nervous System (CNS) and/or induce BBB breakdown remain poorly understood due to difficulties in manipulating the BBB *in vivo*, interspecies differences in animal models, and the limited access to clinical samples [15]. The use of BBB MPS has proven helpful to increase our understanding of the pathogenicity of these microorganisms (Fig 1).

### Cerebral malaria

Around 1% of all symptomatic cases of malaria result in severe disease, of which cerebral malaria is the most complicated manifestation, associated with BBB breakdown and brain swelling [33]. The pathogenesis of cerebral malaria is multifactorial and still not well understood. Histopathology studies consistently find sequestration of *Plasmodium*-infected erythrocytes in the brain microvasculature [34], and recent studies have shown accumulation of immune cells as well [35]. Nevertheless, histopathological analyses of human samples bring their own challenges in terms of the type of research that can be performed, costs, and ethical limitations. Researchers have resorted to studying rodent malaria as a proxy for human disease, but many key mechanisms differ between these two diseases, including its hallmark: infected red blood cell sequestration to the brain vascular endothelium [36].

In recent years, 3D models of both the brain microvasculature and the BBB have been developed to study cerebral malaria [17]. For instance, 3D grid-like perfusable microvasculature models created by soft lithography (see concept in Box 1) recapitulate multiple flow conditions in a single device. 3D microvessels are organ-on-a-chip models of the vasculature: miniature, microfluidic devices that, under controlled flow conditions, mimic the dynamic microenvironment, architecture, and physiological functions of human tissues. The interaction of *P. falciparum* with receptors in brain endothelium is exclusively found in human and great apes and not in rodent experimental cerebral malaria models. Therefore, 3D microvessels have been essential to elucidate several aspects of cerebral malaria, including the effects of differential flow on infected erythrocyte sequestration [37], the dynamics of endothelial inflammatory response mechanisms [38], the roles of BBB cell types in cerebral malaria pathogenesis [16,39], and reproduced the disruption of the angiopoietin-Tie2 axis, a well-known disrupted pathway in patients [39] (Fig 1).

**Box 1. Microfabrication techniques**

**Fig 2:**
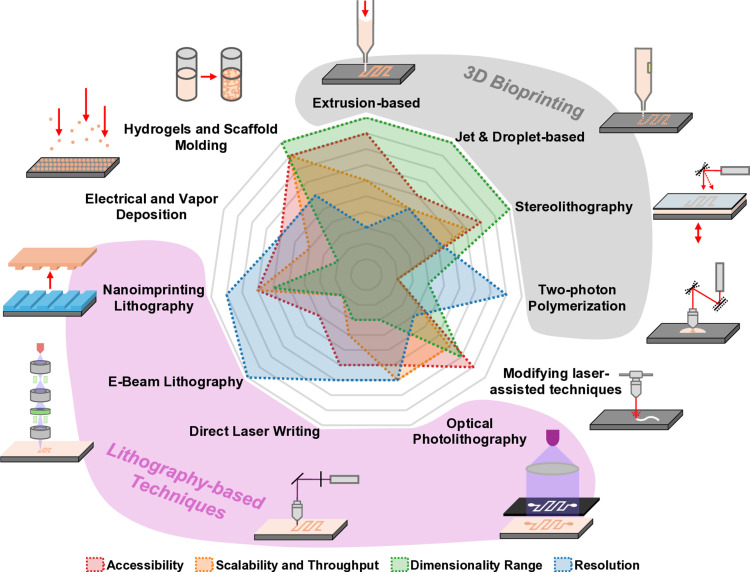
Potential techniques for fabricating MPS in host–parasite research. The radar plot provides a qualitative assessment, encompassing the following factors: (i) accessibility (considering equipment acquisition, maintenance, infrastructure requirements, and technical expertise), (ii) scalability and throughput (ease of production parallelization and scale-up), (iii) dimensionality range (design flexibility across the three spatial axes: X, Y, and Z), and (iv) resolution (the minimum achievable feature size).

Microfabrication is the creation of microstructures and patterns using different techniques. This technology is widely applied in several fields, such as electronics, physics, pharmacology, and medicine [98]. In recent years, new developments have been made in the bioengineering field, namely in the recreation of MPS [3]. Soft lithography stands out as the most widely utilized technique for crafting MPS in host–parasite interaction research. Here, we describe this method alongside a broad spectrum of potential techniques [99] (Fig 2):

- **3D Bioprinting:** Additive manufacturing process, where the materials, known as bioinks, are deposited layer-by-layer to obtain the final 3D construct. Bioinks are composed of hydrogel polymers (natural, synthetic, or composites), cell laden, or fully acellular, and may contain additional bioactive components, such as fibers, minerals, particles, small peptides, growth factors, and extracellular matrix components. While new 3D bioprinting technologies keep regularly emerging [100], the extrusion-based is still the most popular, where the bioink is melted and/or reshaped as filaments via extrusion through a nozzle, deposited, and (if needed) hardened on a building platform. Hardening can be achieved by cooling or via crosslinking (e.g., aerosol). The main limitation of extrusion is size. Alternatively, higher resolutions are usually achieved with droplet-based or material jetting, where the bioink is ejected through a printing nozzle or inkjet head as a droplet or liquid jet, by a thermal or piezoelectric actuator, and sequentially getting cured using a specific wavelength. Other approaches use a laser scan or an image projection to crosslink photosensitive bioinks—a technology known as stereolithography. Depending on the 3D bioprinting technology, the smallest feature size can range between 5 µm and hundreds of micrometers. Two-photon polymerization (2PP) has emerged as a method to create highly precise and intricate microstructures down to a few hundreds of nanometers. 2PP uses two photons absorbed simultaneously by a photosensitive bioink (or other resins) to trigger polymerization at the focal point of the laser.
- **Lithography-based techniques:** Microfabrication of a master mold using a combinatorial workflow of additive, modifying, and subtractive unit processes such as deposition, patterning, and etching, respectively. Photosensitive chemicals, known as photoresists, are homogeneously spread with a defined thickness on a flat substrate, usually glass or silicon wafers, baked, and modified with ultraviolet (UV) or infra-red (IR) light exposure to generate patterns down to sub-micrometer resolution in mask-based (optical photolithography) or maskless (direct laser writing) systems. Scanning a focused beam of electrons (E-Beam Lithography) can be used for features down to just a few tens of nanometers. Final patterns are obtained after immersing the exposed substrate in a developer. Additionally, the substrate or added deposited films sensitive to (dry or wet) etching can be subtracted. Commonly, the master mold is used to generate inverted replicas made of the silicone elastomer PDMS by soft lithography, or made of thermoplastics, such as polystyrene (PS), polymethacrylate (PMMA), or cyclic olefin copolymer (COC), by thermal nanoimprinting lithography, as structured coatings on wafers, or by hot embossing of blocks or film sheets. Photo or UV-based is another variant of nanoimprinting lithography to solidify the resist coating by UV exposure, without requiring heat. As there is a limited number of molding and demolding cycles that can be made, it is often useful to convert the master mold or even the replicas in other types of materials. For instance, using PDMS as stamps to generate patterned matrices of high-performance biocompatible or native polymers.
- **Electrical and vapor deposition:** used to create thin films, coatings, or 3D scaffolds by depositing material onto a substrate. Applying an electric field is possible to deposit polymers, metals, ceramics, or composite materials onto a substrate or mold, forming films or scaffolds with specific physical properties such as porosity, morphology, and electrical conductivity. These methods include electrospinning, electroforming, electrochemical, and electrophoretic deposition. Other procedures rely on generation and deposition of material in the vapor form, typically under a controlled atmosphere. Such methods include Physical Vapor Deposition, Chemical Vapor Deposition, Atomic Layer Deposition, Plasma Spray Deposition, and Vapor Phase Polymerization. Bioactive coatings (e.g., hydroxyapatite, calcium phosphates), particles, fibers, or biomolecules are frequently added to enhance biocompatibility and tailor the mechanical, rheological, and biological biomimicking properties.
- **Hydrogels and scaffold molding:** gelation or liquid-to-solid modifying methods can be used to generate 3D structures inside prefabricated molds or containers. These include sol-gel transition, which involves the conversion of a solution into a gel via a temperature or pH shift (e.g., Poly(N-isopropylacrylamide) (PNIPAAm), chitosan); physical (via ionic or hydrogen bonding) or chemical (via covalent bonding) crosslinking (e.g., glutaraldehyde and calcium ions (for alginate)); and freeze-drying (lyophilization) where water is removed from a hydrogel via freezing and then sublimation, leaving behind a porous, sponge-like structure.
- **Modifying laser-assisted techniques:** Lasers can be used to create microstructures in different materials at a microscopic scale. As the laser energy is absorbed by the material, depending on the material properties and the laser parameters, it is possible to perform operations such as precise and localized melting, sintering, or photoablation (from engraving to a total cut).

A hallmark of severe malaria is microcirculatory obstruction, which occurs in high-density capillaries as narrow as 5 μm in diameter. By combining soft lithography with multiphoton laser photoablation (i.e., ultrafast pulses enabling precise material removal at the focal point without thermal damage of surrounding areas), researchers have been able to create capillary-sized vessels that are later lined by migrating human primary endothelial cells [6]. Plasmodium *falciparum* perfusion of arteriole or venule-sized vessels joined by capillaries of 5–10 μm in diameter showed how infection affects red blood cell speed, deformability and its interaction with the capillary vessel wall, which ultimately exacerbates sequestration and vascular occlusion [6].

### Cerebral animal african trypanosomiasis

Animal African Trypanosomiasis (AAT) is primarily caused by *Trypanosoma congolense*, *Trypanosoma vivax,* and *Trypanosoma brucei,* leading to considerable direct and indirect economic losses [40]. *Trypanosoma congolense*-induced AAT can progress to acute cerebral trypanosomiasis, characterized by increased sequestration to the BBB and lethal neuro-immunopathology [8]. Whilst a mouse model of acute cerebral trypanosomiasis exists and offers views on multiple important factors, including infection outcomes, virulence, drug resistance, and disease tolerance, decoupling immune response-mediated mechanisms from those directly determined by parasite sequestration is challenging to achieve *in vivo*. Recently, a 3D microvessel model that mimics the bovine brain microvasculature has been developed [32]. It offers a cheaper and controlled alternative to study ligand–receptor interactions, the role of biomechanics, and the direct contributions of parasite-intrinsic factors to vascular pathology in the absence of the immune system. It has been useful to assess the contribution of flow velocity, parasite strain, and endothelial cell bed on parasite sequestration in a model of the natural host. The engineering system used to fabricate bovine microvessels is the same as the human, which highlights the versatility of soft lithography generated microenvironments and their applicability to multiple hosts, tissues, and diseases.

### Granulomatous amebic encephalitis

Granulomatous amebic encephalitis (GAE) is a rare but highly fatal human disease that can be caused by free living ameba, such as *Acanthamoeba spp.*, *Sappinia pedata,* or *Balamuthia mandrillaris* [41]. It is endemic worldwide, with ~23,000 cases being reported yearly, mostly in immunocompromised individuals. In these conditions, GAE is a severe infection of the CNS, with more than 90% mortality rate [42]. Current knowledge derives from case reports [43], *in vitro* systems, and mouse models of infection [44].

*Acanthamoeba castellanii* attachment to human BBB under flow has been studied using a monolayer of human brain microvasculature endothelial cells perfused with media containing *A. castellanii* at different flow rates. There were significant differences between binding rates in traditional static monolayer assays and under different flow conditions [45]. Authors also found that secreted amebicidal/parasite proteins may play a role in BBB breakdown during an *A. castellanii* infection. In an alternative system, neurospheroids were successfully used to assess amebicidal activity and neural cytotoxicity against *B. mandrillaris*. Spheroids are 3D cell aggregates that self-assemble in a non-adherent 3D environment and mimic certain aspects of tissue organization and function. They are simpler than organoids and do not require cellular differentiation, which makes them more scalable. These spheroid models were previously developed for cancer studies [46] and later adapted for the study of GAE. Spheroids were grown using human neuroblastoma SH-SY5Y cells and human lung carcinoma A549 cells, in specialized 24-well plates that allow waste drainage, preventing parasite overgrowth. This allowed researchers to overcome the difficulties associated with assessing human cell viability being in the absence of *B. mandrillaris* overgrowth [47]. We did not find additional MPS studies for GAE, which potentially reflects the small size of its research community. The high mortality and low incidence limit clinical data availability, making it harder to design MPS that accurately reflect human pathology. Still, this gap presents a critical opportunity to develop such advanced MPS model to uncover the GAE disease mechanisms, improve drug screening, and bridge the lack of clinical data.

### Toxoplasmosis

*Toxoplasma gondii*, the causative agent of toxoplasmosis, infects around one-third of the human population, and despite being largely asymptomatic, it causes disease in immunocompromised individuals and unborn babies [48]. *Toxoplasma gondii* is highly promiscuous, infecting a broad range of cells [49], and therefore can cause assorted clinical outcomes. It has been recently shown that *T. gondii* interacts differently with different host cell types [50], but further studies are necessary to fully comprehend these host–parasite interaction mechanisms. The use of microenvironments may help in decoding infection mechanisms in distinct environments.

*Toxoplasma gondii* has two stages: tachyzoite, responsible for acute infection, and bradyzoite, a cyst form responsible for latent infection in humans [48]. Using a soft lithography, a recent model that allows the visualization of the crucial stages of *T. gondii* infection of vascular endothelial cells has been developed [18] (Fig 1). With this model, researchers were able to assess in real time the tachyzoite infection of vascular endothelial cells under flow conditions in co-culture with neurons. This system allowed to characterize for the first time a more complete timeline of the events surrounding tachyzoite infection, namely parasite movement and transmission between host cells.

## Models of the cardiovascular system

The cardiovascular system plays a crucial role in parasitic infections, both as a potential microenvironment for infection and a dissemination vehicle. The heart wall is composed of multiple layers: the pericardium, epicardium, myocardium, and endocardium, all of which may be afflicted by several parasitic diseases, including Chagas disease, filariasis, AAT, and toxoplasmosis [51]. The endothelial cell wall lining coronary blood vessels may also be a potential site of infection and subjected to breakdown.

### Chagas disease

Chagas disease, caused by *Trypanosoma cruzi* infection, is a parasitic disease endemic to South America. Upon entering the chronic stage of the disease, 20%–30% of infected individuals develop heart complications (e.g., myocarditis and myocytolysis), commonly known as Chagas cardiomyopathy. Despite comprising the most common cause of death from Chagas disease, the mechanisms behind it remain poorly understood [52].

Recently, MPS have been applied to the study of Chagas cardiomyopathy [31,53]. Here, researchers have opted for 3D cell culture systems that require the seeding of the cells onto a pre-molded microstructure or substrate, allowing them to grow like they would on a natural substrate [54], and assembling into 3D structures, such as spheroids, rods, toroids, and honeycomb or other mesh-like architectures [55]. By seeding cardiomyocytes in agarose coated u-well plates, spheroids that resembled cardiac tissue were created, with spontaneous contractility, cell differentiation, and production of growth factors [31]. Upon *T. cruzi* infection, these spheroids were able to properly develop cardiac fibrosis, enabling further research on this pathology (Fig 1).

### Animal African trypanosomiasis

In *T. congolense*-induced AAT, sequestration occurs beyond the brain, in tissues including the heart, brain, spleen, kidney, and adipose tissue [2]. During chronic disease, parasites can accumulate in the heart and coronary vasculature. In a recent study, researchers have been able to create a bovine cardiac 3D model, using a similar rationale as the model described for cerebral AAT [32] (Fig 1). In this study, these microvessels were seeded with bovine aortic endothelial cells, to mimic the cardiac vasculature. Different parasite strains were shown to adhere differently to different endothelial cell beds and cyclic AMP homeostasis was found to play a critical role in parasite sequestration [32], corroborating what was previously observed in parasite attachment to plastic substrate [56].

## Models of the liver

The liver plays a crucial role in the body’s response to parasitic infections, often serving as a primary site of infection, immune response, and parasite persistence. It is integral to the body’s defense mechanisms and can be significantly impacted by various parasitic diseases.

### Malaria

Inside the mammalian host, *Plasmodium spp.* has two stages: the liver stage and the blood stage (Box 2). The liver stage is an obligatory stage of infection, as parasites need to infect hepatocytes to develop into merozoites, which are then released back into the blood [57]. A human liver platform comprised of hepatocytes and fibroblasts seeded on a micropatterned (see Lithography-Based Techniques section in Box 1) collagen model supports *P. falciparum* and *P. vivax* infection [58] (Fig 1). The same model allowed the characterization of the developing *P. vivax* liver stages, including the analysis of the hypnozoite transcriptome [25]. It also allowed the assessment of relapse frequencies between strains and primaquine clearance. In 2022, this model was used to culture patient-derived *P. vivax* parasites to obtain single-cell transcriptomes for the study of malaria relapse in humans [59].

**Box 2. Summarized life cycles of parasites addressed in this review**

In this review, we mention 12 parasites of human and veterinary relevance: *Plasmodium spp.*, *Trypanosoma spp.*, *Leishmania spp.*, *Toxoplasma gondii*, *Cryptosporidium* spp., *Giardia spp.*, *Acanthamoeba spp.*, *Entamoeba spp.*, *Teladorsagia spp.*, *Strongyloides spp.*, *Schistosoma spp.,* and *Onchocerca spp.*. Here, we provide a summarized life cycle of each parasite type:

- *Plasmodium spp.*: *Plasmodium* sporozoites are transmitted to the human skin by an infected mosquito. Via the bloodstream, they reach the liver, where they invade hepatocytes and divide rapidly, releasing merozoites that infect erythrocytes. Infected erythrocytes can sequester to blood vessels, causing severe pathology. Some merozoites differentiate into gametocytes, which can be ingested by mosquitoes during subsequent blood meals, leading to vector infection and subsequent transmission.
- African trypanosomes: Metacyclic trypanosomes are transmitted by infected tsetse flies to the skin of a mammalian host. The parasites reach the bloodstream, differentiating into bloodstream forms, which multiply in various bodily fluids or tissues, and can be further transmitted to tsetse flies. In the fly, the parasites become midgut procyclic forms and then travel anteriorly to establish in the salivary glands or mouthparts, where they differentiate into epimastigotes and metacyclic forms.
- *Trypanosoma cruzi*: Metacyclic trypomastigotes are transmitted in triatomine bug feces, entering the host through wounds or mucous membranes. They invade a wide range of cells, and differentiate into intracellular amastigotes. Amastigotes differentiate into trypomastigotes as they are released from the infected cell and can infect new cells or be ingested by triatomine bugs. There, they differentiate into epimastigotes in the midgut, multiply, and establish in the hindgut, where they differentiate into metacyclic trypomastigotes.
- *Leishmania spp.*: *Leishmania* promastigotes are injected by a sandfly during a blood meal and are phagocytosed by macrophages. Inside the macrophage, they differentiate into amastigotes and multiply, causing the infected macrophage to rupture and release amastigotes to the bloodstream. At a subsequent blood meal, sandflies can ingest recently released amastigotes or infected macrophages. In the fly gut, amastigotes differentiate into promastigotes and migrate to the proboscis where they remain until transmission.
- *Toxoplasma gondii*: Tissue cysts present in the environment are ingested by a cat or other felines and establish in their intestine becoming fecal oocysts which are released in the feces to the environment. These can be taken up by an intermediate host, such as humans, via sporulated oocysts from contaminated soil, water, or food; undercooked meat containing tissue cysts, or transplacental transmission. Once in the intermediate host, oocysts differentiate into tachyzoites, which spread through the body and differentiate into tissue cyst bradyzoites.
- *Cryptosporidium spp.*: Oocysts exist in contaminated food or water, when they are ingested by the mammalian host, they become active and release sporozoites, which invade gut epithelial cells and differentiate into trophozoites. Trophozoites differentiate into type 1 meront, and then merozoites, which can either enter the asexual replication cycle, differentiating back to trophozoites, or enter the sexual replication cycle, producing microgametes and macrogamonts. Fertilization of these two cells results in a zygote, which leads to thin-walled oocysts for infection of other cells and thick-walled oocysts for transmission via the oral-fecal route.
- *Giardia spp.*: Cysts present in contaminated water, food or feces can be ingested by mammalian hosts. In the small intestine, they excyst, releasing trophozoites, which multiply in the lumen. As the parasites travel toward the colon, trophozoites are encysted and exit the host in their feces.
- *Acanthamoeba spp.*: Entry of *Acanthamoeba* cysts or trophozoites into a mammalian host can occur through the respiratory tract, eye, or skin wounds. When environmental conditions are favorable, trophozoites excyst and divide, when conditions become harsh, trophozoites encyst and become dormant.
- *Entamoeba spp.:* Ingested cysts present in contaminated food or water excyst in the small intestine, releasing trophozoites, which migrate to the large intestine where they proliferate and can invade the intestinal mucosa. Some trophozoites undergo encystation in the lower colon and cysts are released in the feces to the environment.
- *Teladorsagia spp.*: Adult worms in the abomasum (equivalent to the stomach) of the host produce eggs that are passed in the feces. In the environment, eggs hatch into first-stage larvae and develop into second stage and third stage larvae. The latter can remain in the environment for long periods of time until ingested by grazing ruminants. At this stage, larvae penetrate the gastric glands, where they develop into fourth-stage larvae, which can either remain in the mucosa or emerge into the lumen as early adult worms that produce eggs to be excreted.
- *Strongyloides spp.*: Infective filariform larvae enter an intact host’s skin and migrate to the small intestine where they develop into adult female worms. These produce eggs which hatch into rhabditiform larvae in the intestinal mucosa and later migrate into the intestinal lumen, from which they can be excreted in feces to continue the free-living cycle or develop into filariform larvae within the host, leading to autoinfection. In the environment, rhabditiform larvae differentiate into infective filariform larvae to initiate the parasitic cycle in a new host or into free-living adult males and females, which can mate and produce eggs, hatching into rhabditiform larvae.
- *Schistosoma spp.*: Eggs present in contaminated freshwater hatch and release miracidia that swim and penetrate snails. In the snail, they develop into sporocysts and later cercariae, which are released from the snail into water and can penetrate the skin of a mammalian host. In the mammal, cercariae become schistosomulae, which migrate to the lungs, heart, and liver. In the liver, they mature into adult worms, which can mate and migrate to mesenteric venules or bladder venous plexus. Adult females lay eggs that cross blood vessels and are excreted in feces or urine.
- *Onchocerca spp.*: Adult worms live in subcutaneous tissues of the mammalian host and produce microfilariae that migrate to the skin and eyes, and connective tissues. When a blackfly takes a blood meal from an infected person, it ingests microfilariae, which penetrate the fly midgut and migrate to the thoracic muscles, where they develop into larval stages. Infective third-stage larvae migrate to the fly’s proboscis and are transmitted to a new human host in subsequent blood meals.

### Amebic liver abscess

*Entamoeba histolytica* is a protozoan parasite with tropism to the gut, and infection is mostly asymptomatic. However, in around 9% of cases, *E. histolytica* trophozoites may cross the intestinal epithelium and invade the liver, forming amebic liver abscesses (ALA) [60]. Every year, around 40,000–100,000 people die of amebic liver abscesses [61], making it the second leading cause of death among parasitic infections, after malaria.

Whilst animal models of ALA have been successfully used to study key aspects of disease, MPS can be useful to elucidate mechanistic insights that require more controlled microenvironments. In this light, a 3D liver model was developed, which helped to overcome the many constraints behind the use of human samples [62] (Fig 1). This model allowed the study the role of galectins in liver infection. In previous animal models, it was shown that amebic adhesion to host cells was dependent on galactose- or N-acetyl-galactosamine-inhibitable (Gal/GalNAc) lectin, but the use of the 3D model revealed the mechanistic details of this dependence. We did not find additional examples of MPS uses for ALA research, which likely reflects its small field.

## Models of the spleen

### Malaria

The spleen is important for clearance of infected red blood cells and the development of malaria immunity [63]. For some *Plasmodium* species, such as *P. vivax,* the spleen may also act as an infection reservoir, leading to parasite persistence [64].

The spleen is responsible for, among other functions, removing deformed or senescent erythrocytes and other particles, including *Plasmodium spp*., from blood circulation [65]. A process that has been recapitulated in soft lithography-generated microfluidic models [66], which mimicked the complex circulation of the spleen and allowed the study of the biomechanics of *Plasmodium*-spleen interactions. During the spleen filtering process, erythrocytes undergo a process known as “pitting”, after passage through interendothelial slits, all unwanted particles and damaged erythrocytes are removed, including parasites, without damaging the infected host cell. Splenic pitting of malaria parasites has been recreated in an acellular microfluidic device generated by soft lithography [27] in a reductionist model that achieves filtration without compromising cell integrity, showing that immune phagocytosis is not essential for pitting (Fig 1). This illustrates the importance of accurately mimicking tissue mechanical cues, even in the absence of cells and other biological elements.

## Models of the gastrointestinal tract

The gastrointestinal (GI) tract homes many parasitic infections, including helminths, such as *Teladorsagia circumcincta* and *Ascaris Lumbricoides,* and protozoans, such as *Giardia spp.*, *T. cruzi*, *E. histolytica,* and *Cryptosporidium parvum* [67]. The GI tract provides ample nutrition for parasites and may also serve as a gateway for systemic infections, as is the case in the above described ALAs. Examples of 3D gastrointestinal models applied to the study of parasitic diseases are vast.

### Giardiasis

*Giardia lamblia* (i.e., *intestinalis*) is the most common cause of parasitic diarrhea in the world [68] and consequently a public health concern. *G.* *lamblia* attachment to the intestinal epithelium is a crucial step for infection and parasite survival in the host. Although many models have been created to study *G. lamblia* attachment to the gut epithelium, understanding of attachment mechanisms, such as adhesion force under flow, remains elusive [21,69].

Gut-on-a-chip models have primed the discovery of the molecular factors driving parasite attachment to the gut, using similar soft lithography techniques than those highlighted previously in liver and spleen studies. *G.* *lamblia* trophozoites have been cultured inside a perfusable microfluidic chips to evaluate attachment efficiency under different flow rates [69] (Fig 1). The microfluidic network was designed to test in parallel different shear stress levels to simulate the intestinal tract microenvironment. This allowed estimation of adhesion force and determination of EC50 of compounds of interest in a single assay. Although this model did not include host epithelial cells, a previous microfluidic model including an intestinal cell monolayer system was sufficient to assess the role of other factors, such as tonicity, in *G. lamblia* detachment [70].

### Intestinal amebiasis

Intestinal amebiasis is caused by the protozoan parasite *E. histolytica*. It is mainly transmitted through the oral-fecal route and may present some mild to severe symptoms, such as diarrhea, and abdominal pain. In more severe cases, it can progress to peritonitis and be associated with anemia [71].

Polydimethylsiloxane (PDMS)-based microfluidic models can exploit the addition of two lateral vacuum chambers that generate strain and mechanical stretching to mimic intestinal peristalsis. A system like this has been used to assess the effects of mechanical cues on *E. histolytica* intestinal tissue invasion of colorectal adenocarcinoma cells (Caco-2) [19] (Fig 1). This is a recent model, but that has the potential to accelerate fundamental research on intestinal amebiasis and be adapted to other intestinal pathogens.

### Cryptosporidiosis

Cryptosporidiosis is caused by *Cryptosporidium spp*., mainly *C. parvum* and *C. hominis*, transmitted orally through the ingestion of contaminated food and/or water [72]. It is a major cause of diarrheal illness and poses a significant threat to children in developing countries, being one of the leading causes of diarrhea deaths in children under 5 years of age [73].

In Wilke and colleagues [74], an air–liquid interface model was developed that allowed for the full development of *C. parvum in vitro*. With this model, researchers were able to mimic the intestinal microenvironment and assess the gene expression differences across the parasite life cycle. An organoid alternative, capable of supporting the full *C. parvum* development, was later developed [75]. Organoids are 3D miniaturized, self-organizing tissue cultures derived from stem cells that replicate key structural and functional features of real organs. This model involves the formation of mini-gut tubes through laser ablation of a collagen-Matrigel hydrogel and supports long-term *C. parvum* infection [75]. Indeed, one challenge of printed microenvironments is maintaining them long-term, both due to material breakdown and loss of intrinsic cellular characteristics. However, long-term maintenance is crucial to understand parasitic diseases with long infection or incubation times. An early attempt at long-term culturing of *C. parvum* used a 3D human intestinal model containing intestinal myofibroblasts based on a silk scaffold seeded with colorectal adenocarcinoma cells (Caco-2) and goblet cells (HT29-MTX) [20] (Fig 1).

### Helminthiasis

*Teladorsagia circumcincta* is a nematode of significant animal health importance, causing parasitic gastroenteritis in sheep, but available treatments are becoming increasingly ineffective [76], reinforcing the need to develop novel methods to study this helminth and potentially find new drugs [76]. Due to their customization potential, MPS can be useful to gradually increase biological complexity. An example is the integration of naturally occurring gut flora to address host–microbe–parasite interaction. A 3D GI tract model on a collagen hydrogel that couples immune and intestinal cells in co-culture with *Escherichia coli*, a commensal gut bacterium, has been useful to assess the host immune response to *T. circumcincta* and has become a successful proof-of-concept of a complex host–microbe model to study gut–helminth interactions [77].

## Models of the placenta

The placenta is a temporary organ that develops in mammals, which is responsible for supporting the development of the fetus. The placental membrane acts as a selective barrier between the fetus and the mother, allowing for nutrient and oxygen exchange, as well as a layer of protection against infections [78]. Some pathogens can cross or damage the placental barrier [79], but the mechanisms behind many of these interactions remain unknown. Studying placental infection and transplacental crossing is of high clinical relevance, but also highly complex and scarce. It is rare to obtain a placenta at specific stages of development and there are ethical concerns surrounding the available ways to obtain this organ [80].

### Malaria

Pregnant women living in malaria endemic regions are susceptible to placenta malaria, which is characterized by severe complications for the mother, such as anemia and hypertension, and for the babies, including stillbirth or low birth weight [81]. Placental malaria is caused by cytoadhesion of *P. falciparum* infected erythrocytes to placenta trophoblasts through binding to low-sulphated placental chondroitin sulphate A [82]. A recent three-line microfluidic model has been developed, including a channel seeded with human umbilical vein endothelial cells and a third channel including trophoblasts (BeWO cells), both separated by a middle channel containing collagen type I (Fig 1). The use of this model revealed that binding of infected erythrocytes causes a decrease in glucose transport across the placental barrier [30].

### Congenital Chagas disease

*Trypanosoma cruzi* infection during pregnancy results in congenital transmission, as well as significant risk of premature and stillbirth. Although not all parasite strains can be vertically transmitted, the reasons behind these differences remain unknown [83]. In 2024, Apodaca and colleagues used BeWo-based human trophoblasts spheroids seeded on agarose molds to study placental *T. cruzi* infections [29] (Fig 1). Cells exhibited functions resembling *in vivo* conditions, such as proliferation and morphogenesis. Assessment of *T. cruzi* infection of this placental model revealed that infection distribution and progression is strain dependent [29]. Previous attempts to infect spheroids with *T. cruzi* had failed, suggesting reduced susceptibility of JEG-3 cells to *T. cruzi* infection when grown in 3D [84]. This highlights the importance of choosing adequate host cells that can support infection.

## Models of the skin and the underlying fat

Many parasitic infections start in the skin, including leishmaniasis, AAT, malaria, or filariasis. Moreover, the skin can present pathology and serve as a reservoir of infection [23,85].

### Sleeping sickness

The skin can act as a reservoir of *T. brucei* during sleeping sickness [85], so the mechanisms behind parasite survival in the skin are of high relevance. Yet, studying skin-residing trypanosomes *in vivo* is hard for many reasons including the low numbers of infected human patients, the large area of skin in affected animals which challenges trypanosome detection and harvesting, and the invasiveness of acquiring skin biopsies. A high-density skin model obtained through compression of multiple layers of normal primary human epidermal keratinocytes and dermal fibroblasts in collagen was used to study *T. brucei* skin-form parasites [22] (Fig 1). This skin model supports differentiation of natural skin layers and withstands the bite of the tsetse fly (*Glossina morsitans morsitans*). Importantly, this system allowed the characterization of the *T. brucei* population dynamics in the skin: after establishing a proliferative population, *T. brucei* skin-form parasites strongly reduce their replication frequency and metabolic demands, becoming a reversible quiescent population *in vitro*, which may explain chronicity and/or persistence observed in patients [22].

### Schistosomiasis and strongyloidiasis

*Strongyloides ratti* and *Schistosoma mansoni* are nematodes of medical importance: *S. ratti* is often used as an alternative to study human strongyloidiasis and *S. mansoni* is a prominent intestinal parasite causing schistosomiasis [86]. Schistosomiasis has an estimated burden of 2.19 million disability-adjusted life-years and remains a main public health concern [86].

The challenges associated with studying helminth skin forms *in vivo* are similar to those discussed for *T. brucei*. Two models based on primary human epidermal keratinocytes and human dermal fibroblasts grown in a collagen matrix first grown in a liquid–liquid and subsequently in an air–liquid interface were used to study percutaneous *S. ratti* and *S. mansoni* invasion of epidermis [23] (Fig 1). Both parasitic worms invaded a human epidermis equivalent and a human full thickness skin equivalent with similar success rates to *in vivo* studies.

### Onchocerciasis

*Onchocerca volvulus* is the causative agent for onchocerciasis, or river blindness. It is an obligate human parasite, endemic to Africa, with some foci in South America and the Middle East [87]. There are no animal models nor well-established long-term *in vitro* cell culture methods for adult forms [24], and most research relies on the study of similar filarial models, such as cattle *Onchocerca spp*. [88].

MPS may offer an alternative for parasites refractory to standard *in vitro* conditions. Two models have been generated to support cultivation of *O. volvulus* young adult forms, a skin tissue model, made of primary human epidermal keratinocytes and dermal fibroblasts isolated from juvenile foreskin onto a collagen matrix, and an adipose tissue model, made of human bone marrow derived mesenchymal stromal cells seeded onto a u-shaped 96-well plate to form a cell aggregate [24] (Fig 1). This study provides evidence that the microenvironment, specifically the direct contact with cells and ECM, is important for *O. volvulus* development. Further improvement on this model to support full development to mature adult forms is necessary, but larval forms can be harvested from this system for continued culture. An aspect that is particularly important when establishing new culture systems for previously intractable pathogens, such as *O. volvulus*, is to ensure that cultured parasite forms retain their transcriptomic and proteomic profiles, as well as their infectivity and virulence if transferred to an animal host.

## The potential of MPS for translational research

The potential uses of MPS are diverse, and the constant advances made in cell culture and microfabrication techniques enable the development of increasingly complex systems. Under this light, MPS can be used in translational research, by helping to assess drug penetrance in different tissues and their efficacy in various microenvironments, expediting the development of targeted and effective therapies.

### Malaria

Adults living in malaria endemic areas develop immunity to severe disease. The *P. falciparum* parasite ligand that mediates binding to endothelial receptors presents a broad sequence diversity. This argued against the possibility of broadly reactive antibodies. Recently *P. falciparum* binding inhibition studies in a grid 3D microfluidic revealed the existence of monoclonal antibodies that could recognize and prevent the binding of different subsets of parasites with binding affinity to Endothelial Protein C Receptor, one of the main determinants of severe malaria [89]. This study opens the door to study therapeutic strategies to prevent severe disease.

The use of microfluidic models has also been applied for antimalarial research. A multi-organ-on-a-chip model, which combines multiple organ-on-chip models in a single device to recapitulate a system, has been developed for antimalarial efficacy studies [28]. A microfluidic pathway with three chambers was designed and fabricated using laser cutting on clear cast acrylic sheets in combination with PDMS sheets. Each chamber contains different cell types: primary human hepatocytes as a proxy for the liver, human umbilical vein endothelial cells as the vascular endothelium, and primary human splenocyte cells to mimic the spleen (Fig 1). This platform can be used for therapeutic assessment, both drug efficacy and cytotoxicity, where multiple microenvironments are tested simultaneously [28].

### Leishmaniasis

Leishmaniasis is caused by *Leishmania spp.* parasites and has several distinct clinical manifestations. *Leishmania spp*. promastigotes enter the mammalian host via a phlebotomine sandfly bite and are phagocytized by mononuclear cells, such as macrophages. Inside these macrophages, parasites then transform into the amastigote form, where they proliferate [90]. In recent years, the development of new treatment alternatives has become a priority because of increased drug resistance and reduced treatment efficacy. To better study drug efficiency in the treatment of *Leishmania* infected macrophages, there is a need to create a more realistic simulation of drug distribution. In O’Keeffe and colleagues [91], authors assessed several physiologically relevant assays, namely a previously described perfusion-based cell culture model, where infected macrophages are cultured under constant flow [92] and 3D cell culture models, in which infected macrophages are cultured in prefabricated scaffolds. Authors also tested several cell lines and origins of macrophages, namely the use of iPSC-derived macrophages in place of traditional cell lines (human and rodent primary and immortalized cell lines). They used these models and different cell combinations to assess the efficiency of drug uptake by macrophages when perfused with media containing the drugs of interest. This not only served as a comparison study between 3D models and traditional 2D models but also showed that variations in cell types and model constructions directly impact drug activity research, raising awareness on the need of using cell types from multiple donors when assessing drug efficacy.

## Chagas disease

The cardiac spheroid system discuss previously also allowed drug efficacy screening for *T. cruzi*-induced cardiac fibrosis [33]. The effects of posaconazole, a known treatment for fungicidal infections previously shown to have anti-trypanosomal action, were evaluated on the reduction of cardiac fibrosis [53]. As natural cell–cell interactions occur, the spheroid system added a level of accuracy over traditional 2D monolayers used in drug screening. This showed that posaconazole treatment prevented and reduced fibrosis, due to a reorganization of the myocardial tissue.

Despite these examples, the use of MPS for drug development and testing in parasitology is still scarce. Whilst, theoretically, MPS might offer an exciting alternative to animal testing, it remains unclear whether they will truly reach that potential due to both ethical and safety requirements. Nonetheless, in our view, MPS clearly exceed simple culture systems by offering an additional layer of complexity, allowing assessment of the impact of the interactions of different cell types in drug efficacy, and the evaluation of biomechanical parameters in drug delivery and clearance.

## Current challenges of MPS

Throughout this review, we observed that MPS have proven to be versatile tools to study host–parasite interactions. So far, MPS are contributing to our knowledge on tissue-specific tropism, parasite life cycle transitions, culture of intractable parasites, and drug screening. Technical and implementation challenges are being solved: (1) MPS environments often require specialized equipment, biocompatible materials, and trained personnel to design and fabricate, but more and more systems are becoming commercially available; (2) efforts to increase throughput have been made [93]; and (3) while many fabricated microenvironments are short-lived, making them impractical for long term studies, we have discussed a couple of examples where this has been addressed. However, from a biological perspective, many critical aspects remain to be improved if MPS are to become true mimickers of the natural microenvironments. Some of such challenges are the integration of physiological extracellular matrix components; mimicking of circadian rhythms; and accurate modeling of fluid dynamics, particularly in microfabrication. So far, the majority of MPS have understandably relied on a parasite–host tissue vision. However, all these factors directly impact parasite behavior, host cellular responses, and drug efficacy. Therefore, the next big technological challenge is to create fully integrative systems. Perhaps only then MPS will become a complete alternative for animal models of disease. For now, MPS should be tailored to relevant biological questions that can benefit from controlled environments.

## Key knowledge gaps potentially fillable by MPS

Although strong efforts have been made to translate newest developments in bioengineering to the field of host–parasite interactions, MPS still hold unexplored potential to change the way many pathogens are studied. Specifically, we highlight four outstanding gaps to address:

1. **Infection chronicity and persistence**: many parasites remain in the host for prolonged periods of time. Yet, *in vitro* systems that support of long-term infections are scarce. Whilst the system currently developed for *C. parvum* infection [20] holds great potential, it is necessary to invest in this area, as they could expedite the study of long-term drug resistance, experimental evolution, and infection persistence.
2. **Immune evasion mechanisms:** advanced 3D models incorporating immune components could help elucidate how parasites evade or manipulate the host immune response in different tissue contexts, or how an hyperinflammatory response can result in severe disease.
3. **Vector–parasite interactions**: insect cell banks and primary cultures are the main tools for studying vector–parasite interactions. This poses limitations, mainly in terms of achieving biological complexity and in the type of cells that are currently culturable. MPS that mimic insect tissues could provide valuable insights into parasite development and transmission mechanisms, particularly for parasites that undergo complex developmental processes in their vectors before transmission to vertebrate hosts, such as *Leishmania spp*. or *Plasmodium spp*.
4. **Integration of microbiome with parasite–host interactions**: the relationship between pathogenic parasites, host, and microbiome impacts both host health and parasite ecology, so it can have important implications for our understanding of disease mechanisms. Not only the microbiome can act as a “beacon” for parasites, emitting volatile organic compounds that attract or repel them [94], but can also directly affect the type of immune response that is triggered in response to the parasite, as it has been shown for respiratory malaria [95] and intestinal parasitic infections [94,96]. While some progress has been made in developing 3D models of the vertebrate gut that incorporate immune cells and microbiota [97], these models need further refinement to fully capture the intricate interplay between these components and expansion to other parasitic infections.

## Concluding remarks

It is important to achieve multiple levels of resolution, from molecules, to cells, to organisms, to better comprehend how parasites interact with their hosts. There is a wide range of potential applications of MPS in the field of host–parasite interactions, which include the use of microfabrication and cell culture techniques, creation of microtissues, or organ-on-a-chip models, to study an array of aspects behind parasitic diseases, from mechanisms of infection to immunological response to drug efficacy and response studies. Overall, MPS allow a more accurate representation of the parasite’s behavior *in vivo*, providing insights into parasite tropism, virulence factors, and host–parasite co-evolution that may not be observable in traditional systems.
